# Supplementary material for: Identification of photoperiod-induced specific miRNAs in the adrenal glands of Sunite sheep (Ovis aries)
Source: Front Vet Sci. 2022 Jul 22;9:888207. doi: 10.3389/fvets.2022.888207 (PMC9354845; doi:10.3389/fvets.2022.888207)
Supplement: Supplementary file 1 [file Data_Sheet_1.ZIP › Supplementary file/miRDeep2 file.zip!/miRDeep2 file/Novel_Structure/SPLP42A1/Novel_515.pdf]

Provisional ID	:	NC_019483.2_30873
Score total	:	12.1
Score for star read(s)	:	3.9
Score for read counts	:	4.7
Score for mfe	:	1.9
Score for randfold	:	1.6
Score for cons. seed	:	
Total read count	:	21
Mature read count	:	20
Loop read count	:	0
Star read count	:	1

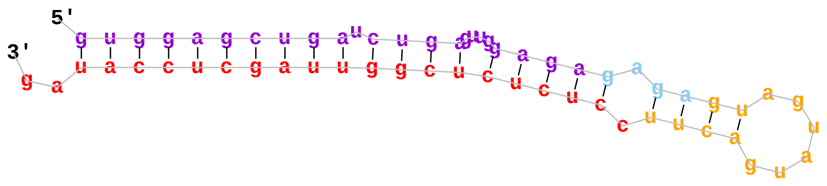

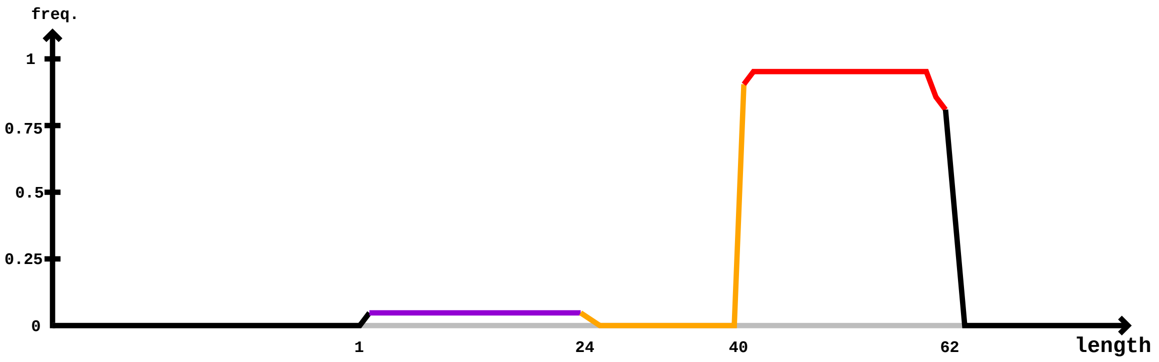

Star **Mature**

```
5'- uuuacucaguucacugaccgauugugccaguguggagcugaucugaguuggagagagaguaguaugacuuccucucucgguuagcuccauagauggcagaucagccuucu  -3'   obs
    uuuacucaguucacugaccgauugugccaguguggagcugaucugaguuggagagagaguaguaugacuuccucucucgguuagcuccauagauggcagaucagccuucu        exp
    .....((((....))))..((((.(((((.(((((((((((.((((....(((((.((((......)))).))))))))))))))))))))..)))))))))........  reads  mm  sample
    ...............................guggagcugaucugaguuggaga........................................................  1      0   seq
    ......................................................................ccucucucgguuagcuccau....................  2      0   seq
    ......................................................................ccucucucgguuagcuccaua...................  1      0   seq
    ......................................................................ccucucucgguuagcuccauag..................  15     0   seq
    ......................................................................ccucucucgguuagcuccauaga.................  1      0   seq
    .......................................................................cucucucgguuagcuccauag..................  1      0   seq
```
